# Advancements in Understanding and Enhancing Antioxidant-Mediated Sperm Cryopreservation in Small Ruminants: Challenges and Perspectives

**DOI:** 10.3390/antiox13060624

**Published:** 2024-05-21

**Authors:** Daniel Ionut Berean, Liviu Marian Bogdan, Raluca Cimpean

**Affiliations:** 1 Department of Reproduction, Faculty of Veterinary Medicine, University of Agricultural Sciences and Veterinary Medicine Cluj-Napoca, Calea Manastur 3-5, 400372 Cluj-Napoca, Romania; liviu.bogdan@usamvcluj.ro; 2 Department of Animal Breeding and Food Safety, Faculty of Veterinary Medicine, University of Agricultural Sciences and Veterinary Medicine Cluj-Napoca, Calea Manastur 3-5, 400372 Cluj-Napoca, Romania; calina-raluca.cimpean@student.usamvcluj.ro

**Keywords:** sperm cryopreservation, antioxidants, small ruminants, reproductive outcomes, cryodamage, fertility

## Abstract

Cryopreservation poses significant challenges to the preservation of sperm integrity and function, particularly in small ruminants where cryodamage is pronounced. This review explores the molecular mechanisms underlying sperm cryodamage and strategies for improving cryopreservation outcomes, with a focus on the role of antioxidants. Cryopreservation-induced alterations in proteins and RNA transcripts critical for sperm function, including motility, capacitation, fertilization, and embryo development, are discussed. Proteomic, transcriptomic, and epigenomic advancements have provided valuable insights into these mechanisms, offering potential biomarkers for predicting sperm freezability and enhancing cryopreservation strategies. Combining technologies such as mass spectrometry and flow cytometry allows for a comprehensive understanding of molecular and cellular changes induced by the freezing–thawing process. However, challenges remain in optimizing cryoprotectant formulations and antioxidant supplementation to improve post-thaw sperm fertility. Further research is needed to explore a wider range of novel cryoprotectants, antioxidants, and proteins for cryopreservation media, as well as to validate their efficacy in enhancing sperm viability and function. Additionally, investigations into the effects of cryopreservation on RNA transcripts and epigenetic factors in small ruminant species are warranted to advance our understanding of sperm preservation. Overall, this review highlights the importance of antioxidants in mitigating cryodamage and underscores the need for continued research to refine cryopreservation protocols and improve reproductive outcomes in small ruminants.

## 1. Introduction

Various external and internal stressors, including the presence of reactive oxygen species (ROS), can adversely affect male fertility. ROS are produced during normal oxygen metabolism primarily as a result of the electron transport chain (ETC) system. This process involves the sequential transfer of electrons through a series of protein complexes, ultimately leading to the formation of ROS. However, under certain circumstances such as altered substrate availability or metabolic changes, there can be a backup in the system, leading to an imbalance between electron donors and recipients. This imbalance can enhance ROS production, as the generated ROS seek alternative targets due to the disrupted flow of electrons, rather than proceeding to molecular oxygen as intended. The pronounced reactivity of ROS allows them to interact with and modify any molecule through oxidation, leading to changes in both structure and function [1,2]. The most prevalent types of ROS produced encompass superoxide anion radicals (O_2_^•−^), hydrogen peroxide (H_2_O_2_), and hydroxyl radicals (^•^OH). The mitochondria are the structures that serve various biological functions reliant on ATP and O_2_^•−^/H_2_O_2_ production, both synthesized through electron transfer reactions. While ATP derives from oxidative phosphorylation, O_2_^•−^ originates from di-oxygen’s singlet electron reduction. Subsequently, superoxide dismutase (SOD) rapidly converts O_2_^•−^ to H_2_O_2_. Formerly regarded as mere by-products of aerobic respiration, O_2_^•−^/H_2_O_2_ now play pivotal roles in cellular processes [3]. 

**Figure 1:**
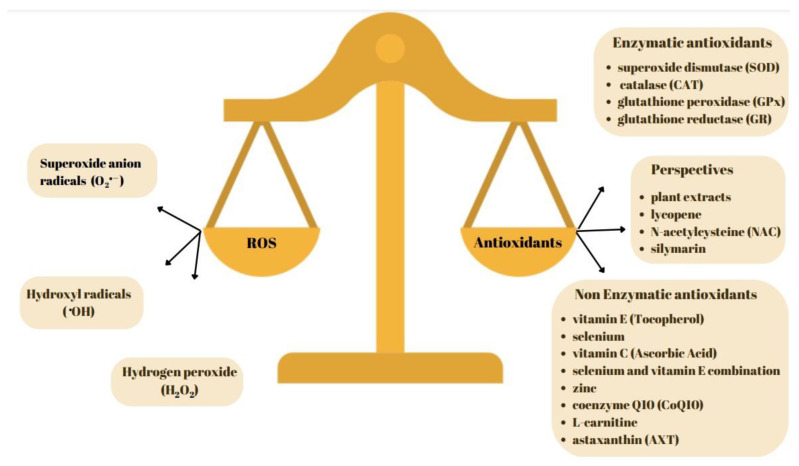
Oxidative status balance in seminal material of small ruminants. On the left side, the reactive oxygen species responsible for spermatozoa fertility decline. On the right side, the main categories of antioxidants used, as well as future perspectives.

Antioxidants play a crucial role in improving the seminal material (sperm quality and quantity) in small ruminants such as sheep and goats. Oxidative stress can negatively affect sperm quality, and antioxidants help combat this by reducing the harmful effects of free radicals and oxidative damage [4]. The relationship between oxidative stress (OS), reactive oxygen species (ROS), and male infertility is indeed complex and multifaceted (Figure 1). Excessive levels of reactive oxygen species (ROS) have been implicated in sperm damage and infertility. However, controlled concentrations of ROS are integral to various physiological processes, including fertilization. It has been observed that numerous transcription factors crucial for tissue development and remodeling are activated by ROS. This may explain the basal ROS production of approximately 2%, even under conditions of optimal mitochondrial efficiency [5]. 

Several antioxidants have been utilized to improve the seminal material in small ruminants, including vitamin E (Tocopherol) [6], selenium [7], vitamin C (Ascorbic Acid) [8], zinc [9], a selenium and vitamin E combination, lycopene, coenzyme Q10 (CoQ10), N-acetylcysteine (NAC), silymarin, and phytochemicals derived from plant extracts [10,11]. These compounds exhibit antioxidant properties and have been shown to enhance sperm quality and protect against oxidative stress. Their supplementation offers potential avenues for mitigating male infertility issues and improving reproductive outcomes in small ruminant breeding programs [12,13].

The aim of this review was to analyze how adding different antioxidants, either at various dilution levels or through general administration, affects the preservation of seminal material in small ruminants. By doing so, the goal was to provide fresh insights for improving protocols in reproductive biotechnologies where seminal material plays a critical role.

## 2. Free Radicals in Small Ruminant’s Semen

Free radicals are highly reactive molecules or atoms that have unpaired electrons. While they play important roles in various physiological processes, an excess of free radicals can lead to oxidative stress, causing damage to cells and biomolecules, including sperm cells [14]. In the context of preserving small ruminant semen, managing free radicals is crucial for maintaining sperm quality during storage. 

Recent reports have highlighted the significant role of reactive oxygen species (ROS) in both reproductive physiology and pathology [2]. The dual nature of ROS is contingent upon factors such as source, concentration, production site, and exposure time [15]. Physiologically, an appropriate level of ROS is deemed crucial for the successful execution of various functions associated with gamete fertility, including proliferation, maturation, the release of oocytes [16], capacitation, hyperactivation, the acrosome reaction, and fertilization [17]. However, excessive ROS production has the potential to induce pathological responses, leading to damage in cells and tissues.

Oxidative stress, resulting from an imbalance between the production of free radicals and the body’s antioxidant defenses, is associated with damage to various molecular components, including lipids, proteins, and nucleic acids. When tissues undergo injury from various factors like trauma, infection, heat, low temperature, toxins, or intense exercise, they can experience short-term oxidative stress. This stress triggers a cascade of events within the tissues. For instance, the injured tissues may upregulate the production of enzymes known to generate radicals, such as xanthine oxidase, lipogenase, and cyclooxygenase. Additionally, they might activate phagocytes, release free iron or copper ions, or interfere with the electron transport chains involved in oxidative phosphorylation. These processes collectively lead to an accumulation of reactive oxygen species (ROS) within the tissues, contributing to oxidative stress [14,18,19]. 

Spermatozoa are highly susceptible to the detrimental effects of reactive oxygen species (ROS) primarily due to the substantial presence of unsaturated fatty acids in their cell membranes. ROS promote the peroxidation of lipids, leading to a loss of membrane integrity and subsequently increasing permeability [20]. Additionally, spermatozoa are particularly vulnerable to oxidative attacks because their cellular membranes contain a high quantity of polyunsaturated fatty acids (PUFAs), which are highly sensitive to lipid peroxidation. Moreover, these cells possess a significant number of mitochondria, which play a crucial role in providing the necessary energy for sperm motility. However, mitochondria also constitute the primary source of ROS in spermatozoa [21]. In spermatozoa, membrane integrity is essential to maintain their fertilizing capacity and to undergo the acrosome reaction [22]. 

The lipid peroxidation of polyunsaturated fatty acids (PUFAs) in spermatozoa results in the impairment of membrane functionality and integrity, and this process varies across different sperm regions [23,24]. For example, when ruminant spermatozoa undergo freezing and thawing, they demonstrate increased lipid peroxidation in the flagellum, which contains higher levels of polyunsaturated fatty acids (PUFAs) compared to the sperm head. This phenomenon is exacerbated by low temperatures and cytotoxicity [24,25]. Malondialdehyde (MDA) is a cytotoxic aldehyde produced as a result of lipid peroxidation, and its measurement serves as a significant marker for oxidative stress (OS) [25,26]. Studies have indicated that the inclusion of antioxidants in the sperm freezing extender can mitigate the destructive effects of reactive oxygen species (ROS) and improve the post-thawing quality of goat and sheep spermatozoa [27].

Spermatozoa proteins play crucial roles in key functions such as sperm motility, capacitation, oocyte binding ability, and the acrosome reaction [28]. Notably, redox-dependent modifications of proteins involve thiol oxidation, tyrosine nitration, and S-glutathionylation. O’Flaherty et al. [28] have reported that these reactions are associated with impaired sperm function, leading to infertility and alterations in sperm motility in ruminants and equines, as well as affecting sperm capacitation. Within proteins, the specific ratio of oxidized to reduced thiol groups (SS/SH) is essential to ensure proper protein functions, but this balance can be affected by ROS [20,29]. Therefore, the quantification of total thiols and/or glutathione (GSH) in cells is critical for monitoring the level of OS. Understanding and assessing these redox-dependent modifications and their impact on protein function provides valuable insights into the mechanisms underlying infertility and sperm dysfunction.

Numerous experiments have consistently provided evidence that both DNA and RNA are vulnerable to oxidative damage, with DNA being identified as a major target [30]. Oxidative nucleotides such as glycol, dTG, and 8-hydroxy-2-deoxyguanosine have been observed to increase during oxidative damage to DNA caused by factors such as UV radiation or free radical damage. Mitochondrial DNA, in particular, has been reported to be more susceptible to oxidative damage, playing a role in the development of various diseases. The identification of 8-hydroxy-2-deoxyguanosine has been suggested as a valuable biological marker for oxidative stress. This molecule can serve as an indicator of the extent of oxidative damage to DNA and is considered useful in assessing the impact of oxidative stress on genetic material. These findings contribute to our understanding of the potential consequences of oxidative damage to nucleic acids and the implications for various physiological and phatological processes [14]. 

## 3. Antioxidants in Small Ruminants Semen Sample

Initially, oxidative stress was simply characterized as the overproduction of ROS. However, in contemporary understanding, it is recognized as a consequence of redox deregulation. The imbalance between the levels of ROS and physiological antioxidant levels can initiate oxidative stress responses. This shift in perspective acknowledges that oxidative stress is not solely defined by the excess production of ROS but is rather a result of disruptions in the delicate balance between oxidants and antioxidants in the cellular environment [31]. 

Sperm cryopreservation induces a significant increase in the production of ROS, leading to various levels of cryodamage. This process poses a challenge as it can compromise sperm quality and fertility. The elevated generation of ROS during cryopreservation (specifically the freezing and thawing processes) can result in oxidative stress, causing damage to sperm cells at the molecular level. This damage may affect various aspects of sperm function, including motility, membrane integrity, and DNA integrity. Managing oxidative stress and employing antioxidant strategies during the cryopreservation process are critical to mitigate the adverse effects on sperm quality and enhance post-thaw fertility potential [32]. 

Antioxidants play a crucial role in neutralizing the overproduction of ROS during sperm cryopreservation. However, if the generation of ROS surpasses the capacity of antioxidants to clear them, or if there is a reduction in antioxidant production, it can lead to a state of OS. In this condition, the balance between ROS production and antioxidant defense is disrupted, resulting in potential damage to sperm cells. Managing this delicate balance is essential in optimizing sperm cryopreservation protocols to minimize oxidative stress and preserve sperm quality [33]. The antioxidant content within sperm is inherently limited, and this can be further reduced during preservation procedures when extenders are used. The dilution of semen with extenders, a common step in sperm preservation techniques, may decrease the concentration of antioxidants present in the sample. As a consequence, the beneficial effects of antioxidants on sperm quality may be compromised. This reduction in antioxidant content during preservation emphasizes the importance of supplementing extenders with exogenous antioxidants. Including antioxidants in the preservation process can help counteract oxidative stress, maintain sperm quality, and enhance the success of sperm preservation techniques. It underscores the need for a careful balance between dilution, which is necessary for storage and transportation, and the preservation of essential components, such as antioxidants, vital for maintaining sperm viability and function [34].

Therefore, the supplementation of antioxidants, even in low concentrations, during sperm cryopreservation represents a promising strategy to optimize these procedures. The potential beneficial effects of these antioxidants on sperm quality in cryopreservation technologies have been extensively characterized and reviewed across various species, including humans, boars, red deer, rams, and others. Research in this area highlights the positive impact of antioxidant supplementation in mitigating oxidative stress, preserving sperm viability, and improving post-thaw sperm functionality. This approach holds great potential for enhancing the success and effectiveness of sperm cryopreservation across different species [35,36,37,38]. 

Living organisms possess natural defense systems, known as antioxidants, which are designed to scavenge and neutralize the effects of ROS. Numerous reports have confirmed the presence of a diverse array of antioxidants in seminal plasma, acting as protective agents against the detrimental effects of ROS [6]. Seminal plasma contains both enzymatic and non-enzymatic antioxidants. Enzymatic antioxidants found in seminal plasma include: superoxide dismutase (SOD), catalase (CAT) and glutathione peroxidase (GPx). Non-enzymatic antioxidants present in seminal plasma include: vitamins A and C, carnitine, glutathione (GSH), and Pyruvate. These antioxidants work collaboratively to maintain a balance in the seminal environment, protecting sperm cells from oxidative damage and contributing to overall sperm health. Understanding and optimizing the antioxidant defense mechanisms in seminal plasma is crucial for preserving sperm quality and fertility [39,40]. 

Under normal conditions, endogenous antioxidant systems are primarily responsible for regulating redox control within the body. However, certain pathological conditions are characterized by an excessive production of ROS, which can overwhelm the endogenous redox control mechanisms. In such circumstances, exogenous antioxidants, obtained from external sources, can play a crucial role in mitigating the detrimental effects of OS. In this context, the focus shifts to the role of exogenous antioxidants in preserving fertility. External sources of antioxidants, such as dietary supplements or antioxidant-enriched media used in assisted reproductive technologies, can contribute to restoring the redox balance, protecting reproductive cells, and potentially improving fertility outcomes. This emphasizes the potential therapeutic value of exogenous antioxidants in situations where endogenous antioxidant defenses are insufficient to counteract the damaging effects of ROS [2].

## 4. The Current Status of Non-Enzymatic Antioxidant Utilization in Enhancing Seminal Material in Small Ruminants

Sperm cells rely on specific antioxidants, such as vitamins E and C, to optimize their viability and fertilization potential. Vitamin E, also known as the anti-sterility vitamin, plays a crucial role in maintaining the normal functionality of the male reproductive system. The susceptibility of sperm cell membranes to oxidative damage is heightened due to the abundance of polyunsaturated fatty acids and insufficient cytoplasmic defense mechanisms. Vitamin E, functioning as a radical scavenger, is essential in mitigating such OS. The regeneration of vitamin E radicals to their active form is facilitated by the presence of vitamin C or ubiquinol. These antioxidants, including vitamin C, E, and ubiquinol, are recognized as chain-breaking antioxidants, effectively neutralizing lipid peroxyl and alkoxyl radicals. Numerous trials have demonstrated the significant role of vitamin E as a primary scavenger of lipid peroxyl and alkoxyl radicals, particularly evident in the protective effects observed during semen cryopreservation. The adverse effects on sperm motility and viability induced by ROS during semen cryopreservation and thawing processes can be attenuated through vitamin E supplementation in extenders. Optimal dosage for such protective supplementation is suggested to be 10 mmol/L, as indicated by various studies [40]. 

Osama Ibrahim Azawi et al. (2013) reported significant (*p* < 0.05) effects of the addition of vitamins C and E to semen diluents on sperm motility and viability at different preservation intervals at 5 °C. They observed significantly higher values of sperm abnormalities and acrosomal defects (37.6 ± 1.3% and 71.5 ± 1.1%, respectively) after 120 h of incubation in Tris-free vitamin C (Control) at 5 °C compared to those containing vitamin C (18.8 ± 1.8% and 52.8 ± 4.3%, respectively). From their findings, it can be inferred that the inclusion of antioxidants such as vitamins C and E in semen preservation media could enhance the longevity and quality of cooled sperm in Awassi ram semen [41]. Also, Chetna Gangwar et al. (2015) demonstrated that the incorporation of vitamin C at a concentration of 56.78 μM could serve as an effective antioxidant in semen diluents during routine freezing procedures, leading to improved post-thaw recovery of buck semen [42]. They reported that semen frozen in diluents containing 45.42 µM and 56.78 µM of vitamin C exhibited significantly improved post-thaw motility and viability (>6%), with the latter concentration also enhancing acrosomal integrity and hypoosmotic swelling positivity. Consequently, the optimal antioxidant level for buck semen diluent was determined to be 56.78 µM of vitamin C, although 45.42 µM also yielded notable improvements in post-thaw motility and live sperm count [42].

The beneficial effects of vitamins C and E on reproductive capacity have also been shown in rams and bucks when added as supplementation to their diets [43,44,45]. Dubing Yue et al. (2010) conducted a study which demonstrated that the supplementation of vitamin E at a concentration of 200 IU/male in sheep diets yielded notable positive effects on both semen quality and quantity (*p* < 0.05). Moreover, this supplementation significantly reduced malondialdehyde (MDA) levels and enhanced the activities of superoxide dismutase (SOD) and glutathione peroxidase (GSH-PX) within the testicular cell membrane and mitochondria (*p* < 0.05) [46]. In another study, Zhu Hong et al. (2010) demonstrated the protective effects of vitamin E supplementation on testicular tissue against oxidative damage in Boer goats. The addition of a dosage of 80 IU of vitamin E per buck led to increased activity of total antioxidant competence (T-AOC) and SOD, while concurrently reducing the content of nitric oxide (NO) within the testicular environment. Supplementation with both low (80 IU per goat) and moderate (320 IU per goat) levels of vitamin E resulted in a decrease in nitric oxide synthase (NOS) activity within the testicular tissue (*p* < 0.05). Additionally, vitamin E administration was associated with an increase in the activity of glutathione peroxidase GSH-PX [47].

On the other hand, despite these positive effects, in a study published in 2023, the authors reported that the antioxidants vitamin C and E did not demonstrate any significant enhancement in semen viability or plasma membrane integrity during the three-day liquid storage period at 5 °C [48]. 

Selenium (Se) plays a crucial role in various physiological functions, particularly in protecting tissues from oxidative damage through its presence in selenoproteins. Se deficiency can compromise antioxidant functions, especially in the testis, affecting sperm quality and reproductive health. In regions with low Se levels in the soil, supplementation becomes essential due to inadequate Se uptake by plants, impacting animal health and reproduction. Previous studies in bucks and rams have shown that selenium supplementation increases plasma GSH-Px concentration, indicating enhanced cellular peroxidase regulation and suggesting improved selenium status. Additionally, selenium supplementation has been found to elevate testosterone levels in Saanen bucks, potentially due to the protective role of GSH-PX against OS in Leydig cells, contributing to enhanced testicular function [49,50,51]. The literature on the in vitro effect of Se on small ruminants is poor; more studies are required to demonstrate its effect. However, Rashid Al-Sarry et al. (2020) reported that the addition of 0.2% selenium nanoparticles to a Tris extender demonstrated a positive impact on most of the ram semen characteristics. These improvements were observed when preserving the semen at 5 °C for 0, 24, 48, and 72 h, as well as for one week post-cryopreservation in liquid nitrogen, indicating potential advancements in artificial insemination techniques in sheep [52]. 

Oral supplementation with sodium selenite for a duration of ninety days yielded notable enhancements in testicular parameters, body weight, and semen characteristics among Saanen bucks. While selenium supplementation notably improved semen quality, particularly evident with the artificial vagina (AV) method of semen collection, the electroejaculation (EE) method did not demonstrate similar improvements. However, the EE method did result in a superior mass and progressive sperm motility. Additionally, selenium supplementation was correlated with increased plasma concentrations of luteinizing hormone (LH) and testosterone, alongside augmented activity of GSH-PX [49]. 

Zinc exerts its influence on reproductive characteristics through the activation and maintenance of the germinal epithelium of seminiferous tubules, as well as by stimulating the production and secretion of testosterone, thereby impacting spermatogenesis. In ram lambs, a diet supplemented with 17.4 and 32.4 mg Zn/kg feed significantly enhanced testes development and sperm production [53,54]. Also, it was reported that a Zn and Se combination could improve semen characteristics which may result in overall improvement in the reproductive performance of Sanjabi rams during the breeding season [53]. 

Lycopene, a potent natural antioxidant, belongs to the tetraterpene carotenoid family and is characterized by its red pigmentation and its thirteen linear double bonds. Lycopene stands out as the most effective anti-radical compound among carotenoids, underscoring its biological significance [55,56]. Research indicates that lycopene exhibits double the effectiveness of β-carotene and ten times that of α-tocopherol [10]. Its lipophilic nature allows for its presence in various sources such as tomatoes, papayas, watermelons, apricots, pink grapefruits, and rosehips [10,57]. Numerous studies have demonstrated the potential effects of lycopene supplementation on seminal characteristics in humans [58,59,60], cattle [61,62], and rats [57,63]. In rams, Souza H.M. et al. (2019) investigated the impact of lycopene supplementation in a Tris–egg yolk extender on various sperm parameters including sperm kinetics, plasma and acrosomal membrane integrity, intracellular ROS production, and lipid peroxidation of the plasma membrane post-freezing and thawing. The study revealed that while the addition of lycopene to the Tris–egg yolk extender during ram semen cryopreservation did not influence the integrity of plasma and acrosomal membranes, intracellular ROS production, or lipid peroxidation of the cell membrane, it did affect progressive motility of cryopreserved spermatozoa after a two-hour incubation at 37 °C, particularly at concentrations of 0.1 µM, 1 µM, and 5 µM [64]. 

Coenzyme Q10 plays a crucial role in improving seminal characteristics by acting as a potent antioxidant, safeguarding sperm cells from oxidative damage [65,66]. Supplementation of 2 μM coenzyme Q10 in the extenders has been demonstrated to enhance sperm motility, viability, and overall semen quality in small ruminants, thereby augmenting reproductive performance [67]. Studies indicate that coenzyme Q10 supplementation improves mitochondrial function in sperm cells, leading to increased energy production and enhanced sperm movement, which are essential for successful fertilization [68].

Sohail T. et al. (2024) demonstrated that supplementation of an optimal concentration of astaxanthin (AXT) at 3.5 µM in Hu ram semen preservation markedly enhances its kinematic properties, longevity, plasma membrane integrity, acrosome integrity, total antioxidant content, and mitochondrial membrane potential. Conversely, the inclusion of AXT at 3.5 µM significantly diminishes ROS and MDA concentrations in spermatozoa, thereby extending their longevity and ultimately enhancing their fertilization potential [69,70]. AXT (C40H52O4) is a red carotenoid pigment sourced from the alga hematococcus pulvalis, renowned for its antioxidant, anticancer, anti-diabetic, and anti-inflammatory properties. Widely distributed in marine organisms such as crustaceans, microalgae, and salmon, AXT has garnered significant attention in recent scientific endeavors [71,72]. Contemporary research reveals that the inclusion of AXT enhances sperm motility, viability, and membrane integrity across various species including bulls, boars, humans, and roosters during semen preservation [73,74,75,76]. Notably, astaxanthin’s unique ability to permeate biological membranes facilitates the protection of fatty acids and cellular membranes from the detrimental effects of lipid peroxidation injuries [77]. Comparative studies highlight astaxanthin’s superiority as an antioxidant over lutein and β-carotene, with antioxidant properties ten times greater than other carotenoids and a hundred times higher than α-tocopherol [78].

Amino acids serve as versatile tools in bolstering sperm resilience against the detrimental effects of cryopreservation-induced cold shock. L-carnitine (LC), a water-soluble amino acid known chemically as (3R)-3-hydroxy-4-(trimethylamine)butanoate and derived from lysine, facilitates the entry of long-chain fatty acids into mitochondria, thereby enhancing sperm motility [79,80,81]. Produced primarily in mammalian epididymal tissue and transported to sperm, increased concentrations of LC at the epididymal level have been associated with heightened sperm motility. Additionally, LC exhibits antioxidant properties, mitigating oxidative stress-induced DNA damage as evidenced by in vivo and in vitro studies [82]. N-acetyl cysteine (NAC), another thiol-containing compound, possesses potent antioxidant capabilities. Serving as a precursor to L-cysteine, NAC aids in scavenging free radicals by reacting with ROS, thereby compensating for decreased glutathione levels during OS. In the context of sperm cryopreservation, NAC prevents the sedimentation of membrane proteins and increases their abundance following exposure to cold shock. Moreover, NAC acts as an antioxidant against ROS activity, effectively safeguarding sperm functionality. Numerous investigations have underscored the beneficial effects of NAC supplementation in cryopreservation media, resulting in enhanced sperm functional parameters [83]. In humans, NAC and LC exhibit remarkable abilities to mitigate OS levels and decrease the production of harmful byproducts, intracellular ROS, and subsequent DNA damage. This reduction in OS translates to the restoration of all sperm functional parameters that may have been adversely affected or damaged by these products within the cryopreservation media. Notably, the protective and beneficial effects of NAC and LC extend to key sperm quality indicators such as the progressive motility index, mitochondrial membrane potential parameters, and various sperm motility characteristics. As such, the inclusion of NAC and LC holds promise for improving sperm function within cryopreservation media, offering potential avenues for enhancing the success of assisted reproductive technologies [82]. In animal studies, the effects of NAC and LC are relatively understudied; however, there are research investigations that have demonstrated their beneficial effects in improving reproductive function and seminal characteristics following dilution and preservation [84,85,86]. In small ruminants, it was reported that moderate concentrations of NAC, such as 0.5 mM and lower, incorporated into skim milk-based extenders, offer a protective mechanism for ram sperm cells against OS without adversely affecting the freezability of ram semen (Table 1); conversely, higher concentrations of NAC, specifically at 0.75 mM and above, particularly when utilized with milk-based extenders, may exhibit some deleterious effects on the freezability of ram semen [87].

## 5. The Current Status of Enzymatic Antioxidant Utilization in Enhancing Seminal Material in Small Ruminants

Enzymatic antioxidants, such as superoxide dismutase (SOD), catalase (CAT), glutathione peroxidase (GPx), and glutathione reductase (GR), are large molecules that safeguard cells from ROS damage. They achieve this by catalyzing reactions that either directly neutralize ROS or regenerate other antioxidants, thereby preserving cellular integrity. These enzymes play a critical role in maintaining redox balance and protecting cells from OS-induced injury. Several studies have explored the supplementation of enzymatic antioxidants in the diets of small ruminants to enhance semen quality and reproductive performance. These studies have investigated various aspects such as sperm motility, morphology, viability, and OS levels [88].

Variations in SOD activity have been noted in relation to semen quality and storage conditions. These fluctuations may stem from OS, where either excessive enzyme use occurs to safeguard sperm quality, or the enzymes are insufficient to maintain it [89,90]. Incorporating SOD into extenders has shown positive outcomes, with concentrations of 800 U/mL or 150 μM providing enhanced protection, particularly for motility, in refrigerated ram sperm cells. Additionally, enhanced pregnancy rates have been reported in ewes inseminated with semen treated with a combination of SOD (800 U/mL) and CAT (200 U/mL), stored for up to 14 days, compared to controls. These findings underscore the potential benefits of supplementing extenders with enzymatic antioxidants for preserving semen quality and reproductive success in small ruminants [91].

Catalase (CAT) has been employed as a supplement to enhance semen’s antioxidant capability and maintain sperm functionality. Studies indicate that adding CAT at concentrations of 100 and 200 U/mL to extenders can mitigate the detrimental impact of cooling on total motility and ram sperm survival during liquid storage at 5 °C. However, caution is warranted as concentrations exceeding 200 U/mL of CAT were found to be toxic to sperm. This underscores the importance of optimizing CAT concentrations to ensure its beneficial effects on semen quality while avoiding potential harm to sperm viability [92,93].

Glutathione peroxidases (GPx) and associated enzymes involved in glutathione (GSH) synthesis and reduction play pivotal roles in cellular peroxide regulation. GPx catalyzes the conversion of hydrogen peroxide to water and lipoperoxides to alkyl alcohols through GSH reduction. Glutathione reductase (GSR) subsequently regenerates GSH from its oxidized form (GSSG), particularly under OS conditions. Despite its non-enzymatic nature, GSH acts as a coenzyme, demonstrating significant effects in semen preservation [34,94]. Supplementation of ram semen extenders with 1–2 mM GSH has been shown to improve sperm viability and reduce free radical levels after prolonged chilled storage (Table 2). Recent research has reaffirmed these benefits during cooled storage at 5 °C for up to 72 h. Findings suggest that optimal concentrations of 200 mM GSH mitigate declines in sperm motility, membrane integrity, motion parameters, mitochondrial activity, and hexose transporter abundance, thus enhancing antioxidant capacity and energy metabolism in stored ram spermatozoa [36,95].

## 6. A Perspective in Exploring Various Antioxidants to Facilitate the Improvement of Sperm Quality in Rams and Bucks

Cryopreservation induces alterations in proteins and RNA transcripts crucial for sperm functions, impacting sperm motility, capacitation, fertilization, and embryo development. Understanding these molecular changes is vital for preserving sperm integrity and enhancing reproductive performance in small ruminants, which are particularly susceptible to cryodamage. Proteomic, transcriptomic, and epigenomic advancements offer valuable insights into the mechanisms underlying sperm cryodamage and factors affecting cryotolerance [38,96]. Combining technologies like mass spectrometry and flow cytometry can provide a comprehensive understanding and facilitate the development of improved cryopreservation strategies. However, challenges remain, necessitating further research on novel cryoprotectants and antioxidants to optimize freezing protocols and ensure post-thaw fertility. Comprehensive investigations into the effects of cryopreservation on RNA transcripts and epigenetic factors are also warranted to advance our understanding of sperm preservation in small ruminants.

The use of plant extracts in semen cryopreservation of small ruminants has garnered increasing interest due to their antioxidant properties and potential to mitigate cryodamage. Studies suggest that plant extracts rich in polyphenols, flavonoids, and other bioactive compounds possess antioxidant properties that can protect sperm from oxidative stress-induced damage during cryopreservation. Furthermore, these extracts may enhance sperm motility, viability, and fertility post-thawing [97,98]. However, variations in efficacy and optimal dosage exist among different plant extracts, highlighting the need for further research to elucidate their mechanisms of action and refine cryopreservation protocols. Integrating plant extracts into semen extenders holds promise for improving the success rates of assisted reproductive technologies in small ruminants while also offering a sustainable and natural approach to preserving semen quality.

Lycopene, a carotenoid compound abundant in various fruits and vegetables, has garnered attention for its antioxidant properties and potential benefits in semen cryopreservation. Studies suggest that lycopene’s antioxidant activity may protect sperm from OS-induced damage during cryopreservation, leading to improvements in post-thaw sperm motility, viability, and fertility [58,59,61]. Additionally, lycopene’s ability to modulate cellular signaling pathways involved in sperm function underscores its potential as a valuable additive in semen extenders. However, further research is needed to elucidate the optimal dosage, timing, and mode of lycopene supplementation, as well as its interactions with other cryoprotectants. Integrating lycopene into semen cryopreservation protocols offers a promising avenue for enhancing reproductive technologies in small ruminants while promoting sustainable and natural approaches to semen preservation.

Despite the promising findings about NAC, further research is needed to optimize NAC supplementation protocols, determine the optimal dosage, and assess its long-term effects on sperm functionality and reproductive outcomes. Integrating NAC into semen cryopreservation protocols offers a promising avenue for enhancing reproductive success in small ruminants while promoting sustainable breeding practices.

Silymarin, a natural flavonoid complex derived from milk thistle (Silybum marianum), has gained attention for its potential role in improving semen cryopreservation outcomes in small ruminants. Studies suggest that silymarin’s antioxidant properties may protect sperm from oxidative damage during cryopreservation, leading to enhanced post-thaw sperm quality and fertility [99,100]. Additionally, silymarin’s anti-inflammatory and membrane-stabilizing effects may further contribute to its beneficial effects on semen preservation. However, challenges such as variability in silymarin composition, optimal dosage determination, and interactions with cryoprotectants need to be addressed. Further research is warranted to elucidate the specific mechanisms underlying silymarin’s protective effects on sperm and to optimize its application in semen cryopreservation protocols for small ruminants. Integrating silymarin into semen extenders holds promise for improving reproductive success and promoting sustainable breeding practices in small ruminant production systems.

The majority of existing studies have largely focused on the antioxidant effect of various substances in vitro, analyzing spermatozoa before and after different periods of post-conservation time. However, future research studies are critically needed to validate these experiments in vivo. Research directions should largely address achieving pregnancies following the use of enhanced seminal material in various biotechnologies in reproduction. This approach would allow for the assessment of the real effectiveness of antioxidant substances in improving sperm quality and reproductive success under conditions closer to natural settings.

The stringent selection process for sperm underscores the need for research to prioritize achieving outcomes like pregnancies or embryos. These studies are essential for validating the effectiveness of antioxidant compounds and establishing optimal concentrations. By focusing on attaining tangible results, researchers can advance reproductive biotechnologies and enhance reproductive success in settings more closely resembling natural conditions. This approach ensures that interventions aimed at improving sperm quality translate into meaningful outcomes, ultimately benefiting both animal welfare and agricultural productivity.

## 7. Conclusions

From the extensive body of research focused on antioxidants and their role in enhancing seminal material in small ruminants, several key conclusions can be drawn:

- Enzymatic antioxidants, such as SOD, CAT, and GPx, play crucial roles in protecting sperm from oxidative damage, thereby improving semen quality.
- Supplementation of extenders with antioxidants, including SOD, CAT, and N-acetylcysteine (NAC), has shown promise in mitigating cryodamage and preserving sperm viability and motility during cryopreservation.
- Natural antioxidants, such as those derived from plant extracts like silymarin, offer potential alternatives for enhancing semen cryopreservation outcomes in small ruminants.
- Future research efforts should prioritize in vivo validation of antioxidant interventions and focus on achieving tangible reproductive outcomes, such as pregnancies and offspring, to assess the real-world efficacy of antioxidant supplementation.
- Additionally, studies exploring the optimal dosage, timing, and interactions of antioxidants with cryoprotectants are needed to refine semen cryopreservation protocols and improve reproductive success in small ruminants.

Overall, the integration of antioxidants into semen cryopreservation protocols holds significant promise for advancing reproductive biotechnologies and enhancing the efficiency and sustainability of small ruminant breeding programs.

## Figures and Tables

**Table 1:** Administration routes, dosages, effects, and mechanisms of action of various non-enzymatic antioxidants in the cryopreservation of semen in small ruminants.

Antioxidants	Administration Route, Dosage	Effect	Bibliographical Source
Vitamin E	Addition to extender 10 mmol/L	Significantly higher values of sperm abnormalities and acrosomal defects (37.6 ± 1.3% and 71.5 ± 1.1%)	[41]
	Supplementation of vitamin E at a concentration of 200 IU/male in sheep diets	Positive effects on both semen quality and quantity (*p* < 0.05), reduced MDA levels and enhanced the activities of SOD and GSH-PX within the testicular cell membrane and mitochondria (*p* < 0.05)	[46]
	Addition of a dosage of 80 IU of vitamin E per buck	Increased activity of T-AOC) and SOD, while concurrently reducing the content of NO within the testicular environment, increased the activity of GSH-PX	[47]
Vitamin C	Incorporation of vitamin C at a concentration of 56.78 μM in semen extenders	Significantly improved post-thaw motility and viability (>6%), also enhancing acrosomal integrity and hypoosmotic swelling positivity	[42]
Selenium	Addition of 0.2% selenium nanoparticles to a Tris extender	positive impact on most of the ram semen characteristics	[52]
	General selenium supplementation/ Oral supplementation	Increased plasma GSH-Px concentration, elevated testosterone levels in Saanen bucks, enhanced testicular function, increased plasma concentrations of luteinizing hormone (LH) and testosterone	[49,50,51]
Zinc	Diet supplemented with 17.4 and 32.4 mg Zn/kg feed in ram lambs	Enhanced testes development and sperm production	[53,54]
Lycopene	Lycopene supplementation in Tris–egg yolk extender at concentrations of 0.1 µM, 1 µM, and 5 µM	Increased progressive motility of cryopreserved spermatozoa after a two-hour incubation at 37 °C	[64]
Coenzyme Q10	Supplementation of 2 μM coenzyme Q10 in the extenders	Enhanced sperm motility, viability, and overall semen quality in small ruminants	[68]
AXT	Supplementation of an optimal concentration of astaxanthin (AXT) at 3.5 µM in Hu ram semen preservation	Diminishes ROS and MDA, enhances kinematic properties, longevity, plasma membrane integrity, acrosome integrity, total antioxidant content, and mitochondrial membrane potential	[69,70]
NAC	Moderate concentrations of NAC, such as 0.5 mM and lower, incorporated into skim milk-based extenders	Offers a protective mechanism for ram sperm cells against OS without adversely affecting the freeze-ability of ram semen	[87]

**Table 2:** Administration routes, dosages, effects, and mechanisms of action of various enzymatic antioxidants in the cryopreservation of semen in small ruminants.

Antioxidants	Administration Route, Dosage	Effect	Bibliographical Source
SOD	Incorporating SOD into extenders with concentrations of 800 U/mL or 150 μM	Enhanced protection, particularly for motility, in refrigerated ram sperm cells.	[91]
Combination of SOD and CAT	SOD (800 U/mL) and CAT (200 U/mL)	Increased pregnancy rates	[91]
CAT	CAT at concentrations of 100 and 200 U/mL to extenders	Mitigated the detrimental impact of cooling on total motility and ram sperm survival during liquid storage at 5 °C	[92,93]
GSH	Supplementation of ram semen extenders with 1–2 mM GSH	Improved sperm viability and reduced free radical levels after prolonged chilled storage	[95]
